# Combined Lifestyle Factors and Risk of All-Cause and Cause-Specific Mortality Among Participants in the Linxian Nutrition Intervention Trial: A Cohort, Observational Study

**DOI:** 10.3389/fcvm.2022.772617

**Published:** 2022-02-10

**Authors:** Jin-hu Fan, Jian-bing Wang, Huan Yang, Sanford M. Dawsey, Philip R. Taylor, You-lin Qiao, Christian C. Abnet

**Affiliations:** ^1^Department of Cancer Epidemiology, National Cancer Center/National Clinical Research Center for Cancer/Cancer Hospital, Chinese Academy of Medical Sciences and Peking Union Medical College, Beijing, China; ^2^Department of Epidemiology and Biostatistics, National Clinical Research Center for Child Health, The Children's Hospital, Zhejiang University School of Medicine, Hangzhou, China; ^3^Metabolic Epidemiology Branch, Division of Cancer Epidemiology and Genetics, National Cancer Institute, Rockville, MD, United States

**Keywords:** combined risk factors, mortality, prospective study, linxian nutrition intervention trial, China

## Abstract

**Background:**

Several studies have indicated that combinations of lifestyle and dietary factors are associated with risk of total mortality and death from cardiovascular disease and cancer, but limited data are available from long-term follow-up studies in China.

**Methods:**

This study was a observational cohort study. We prospectively examined the associations of combined lifestyle factors and risk of total and cause-specific mortality in the Linxian General Population Nutrition Intervention Trial (NIT) cohort that included 29,584 healthy adults. A points system method was used to calculate a combined risk score of five lifestyle factors, including smoking, alcohol drinking, body mass index, vegetable intake and fruit intake. Cox proportional hazards models were used to estimate hazard ratios (HRs) and 95% confidence intervals (95% CIs).

**Results:**

Overall, adjusted hazard ratios for mortality increased progressively with an increasing combined risk score. Compared to individuals with a score of zero or one, HRs (95%CIs) for a score of five or above were 1.59 (1.44–1.75) for all-cause mortality, 1.67 (1.48–1.88) for heart disease, 1.69 (1.52–1.88) for stroke, and 1.34 (1.21, 1.47) for cancer. This association for mortality was seen consistently, regardless of gender and age at baseline.

**Conclusions:**

A higher combined risk score was positively associated with risk of total, heart disease, stroke, and cancer mortality. These findings could provide further evidence for the idea that healthy lifestyle is the optimal way to reduce the risk of premature death, and encourage behavior change.

## Introduction

A large body of evidence has suggested that lifestyle-related factors are associated with a higher risk of multiple chronic diseases and premature death (1–6). However, limited studies have examined the combined effects of lifestyle-related factors on risk of mortality (7–15). Quantifying the magnitude of effects of lifestyle-related factors on risk of mortality individually and in combination will help identify priorities for clinical and public health efforts. A previous prospective cohort study of 74,942 women in the Shanghai Women's Health Study indicated a 33% reduction in all-cause mortality and a 59% reduction in cardiovascular mortality among women with four to five healthy factors (including never exposed to spouse's smoking, lower waist-hip ratio, daily exercise, normal weight, and higher daily fruit and vegetable intake), as compared with women with none of these healthy factors (7). Other prospective cohort studies, such as the Nurse's Health Study (13), the EPIC-Norfolk Study (14) and the JACC study (10), similarly reported an inverse association between combinations of healthy lifestyle factors and all-cause mortality. However, most studies for the association of combined lifestyle factors with risk of mortality have been conducted in the Western countries. Little evidence is available from long-term follow-up studies in the Chinese population, whose lifestyles may differ from the Western populations.

The Linxian General Population Nutrition Intervention Trial (NIT) prospectively collected data on all-cause mortality outcomes. The selected lifestyle factors included body mass index (BMI), smoking, alcohol drinking, vegetable intake and fruit intake. Herein we reported the association of the combination of these lifestyle factors and risk of all-cause and cause-specific mortality over 30 years of follow-up in the NIT Cohort in China.

## Methods

### Study Population

This study was a observational study based on NIT cohort. The design of the NIT study has been described previously (16, 17). Briefly, the NIT enrolled healthy individuals between the ages of 40 and 69 years from four communes in northern Linxian. All participants have provided written informed consent. Finally, a total of 29,584 subjects were randomly assigned to one of eight intervention groups which received daily vitamin/mineral supplement combinations or placebo according to a one-half replicate of a 2^4^ fractional factorial design. The intervention began in March 1986 and lasted for 5.25 years.

We excluded participants (*n* = 133) with missing values of five included lifestyle factors. Finally, a total of 29,451 subjects were included in the analysis (13,129 men and 16,322 women) (Supplementary Figure 1).

This study was approved by the Institutional Review Boards of the US NIH and the Chinese Academy of Medical Sciences. All participants gave informed consent for the use of their data.

### Data Collection

Demographic characteristics and lifestyle factors at baseline were collected using a self-designed questionnaire. All participants had their basic physical examination by trained staff using a standard protocol. Body weight and height were measured while subjects were not wearing shoes. Body mass index was then calculated as weight in kilograms divided by height in meters squared (kg/m^2^). Smoking was defined as regular cigarette or pipe use for at least 6 months (including ever or current smokers), and alcohol use was defined as any alcohol consumption in the past 12 months (including ever or current alcohol drinkers). For vegetable and fruit intake, participants were asked how often, on average, they consumed during the last year before the interview date. The possible responses ranged from “never” to “several times per day.” The responses were then converted to different units according to the distribution of our data, e.g., times per week for vegetables and times per year for fruit. Family history of cancer was considered positive if participants reported a cancer in at least one first-degree relative.

### Follow-Up and Outcome

During the trial period (1986–1991), village doctors visited all participants monthly, and all endpoints were confirmed by an International Endpoints Review Committee consisting of American and Chinese experts in cytology, pathology, surgery, and radiology. In the post-trial follow-up (after 1991), village doctors continued to contact all living participants monthly, and new cancer cases and all-cause deaths were verified by a panel of American and Chinese experts (1991–1996) or senior Chinese doctors (1996–2018). Diagnostic materials included case records, pathology and cytology slides, X rays, biochemical results, and ultrasound, endoscopy and surgery reports. Death outcomes were examined with death registration quarterly. The causes of death were coded according to the International Disease Classification Codes, version 10 (ICD-10). Cause-specific mortality included heart disease mortality, stroke mortality, and cancer mortality. Heart disease mortality were defined as death caused by coronary heart disease, hypertensive heart disease, rheumatic heart disease, pulmonary heart disease, or other cardiovascular disease.

### Statistical Analysis

Frequencies and percentages of demographic and other participant characteristics were calculated by gender. Participants were censored at the date of death, last known follow-up date, or January 31, 2018, whichever occurred first.

Two combined risk scores of five lifestyle factors were created to examine the combined effects of the individual risk factors. A combined risk score 1 (CRS1) was created using a points system method based on the selected risk factors. This method was originally developed by Sullivan et al. to evaluate the combined effects of several risk factors on chronic diseases (18). Briefly, we created the CRS1 through the following three steps: (1) running the multivariable Cox regression model that includes all baseline characteristics and selected lifestyle factors, and defining the regression coefficient of age (β_0_) as the constant, representing the regression coefficient for one-year increase in age with risk of mortality; (2) calculating the individual risk point for each level of each lifestyle factor by dividing the respective regression coefficient (β_i_) with the constant (β_0_); (3) rounding the risk points to the nearest integers and calculating the CRS1 by summing individual risk points for each level of each risk factor. We also created a CRS2 by summing the number of five risk factors (total score: range of 0–5 points). Subjects were assigned one point for having the unhealthy factor and zero point for having the healthy factor (more details see Supplementary Table 1).

Cox proportional hazards regression models were used to calculate hazard ratios (HRs) and 95% confidence intervals (95% CIs) for the associations between the individual lifestyle factors / CRS and risk of all-cause and cause-specific mortality. CRS1 were treated as both continuous and categorical variables (CRS1 ≤ 1 as the reference group, based on the distribution) in the Cox models. Proportional hazards assumption was tested by including an interaction term between time and risk factor (*P*>0.05 for all tests) in the Cox models. Potential confounders included age at baseline (continuous variable), sex (men or women), commune (Rencun,Yaocun, Hengshui or Donggang),education level (never, <5 years, primary school, high school or higher education, or other), and family history of cancer (positive or negative). We also performed sensitivity analyses by excluding individuals who were followed up <3 years.

All statistical analyses were performed using SAS software (version 9.4, SAS Institute Inc. Cary, NC, USA). All tests were two-sided and associations were considered significant for *p* < 0.05.

## Results

During 629,144 person-years of follow-up, we identified 20,466 deaths, including 5,026 from heart disease, 6,564 from stroke and 5,996 from cancer. Approximately 1% of participants were lost to follow-up. Table 1 shows characteristics of the participants at baseline by men and women. Women were more likely to be older, less educated (higher education, 16.0 vs. 3.7%), and less commonly smokers and alcohol drinkers.

**Table 1:** Baseline characteristics in the Linxian General Population Nutrition Intervention Trial Cohort.

**Lifestyle factors**	**All subjects**	**Men**	**Women**
No. of participants, *n* (%)	29,451 (100.0)	13,129 (44.6)	16,322 (55.4)
Age [Mean (SD), years]	51.9 (8.9)	52.6 (9.0)	51.3 (8.7)
Person-years	629,144.1	258,773.4	370,370.7
BMI [Mean (SD), kg/m^2^]	22.0 (2.5)	21.7 (2.1)	22.2 (2.7)
**Smoking**, ***n*** **(%)**			
No	20,557 (69.8)	4,274 (32.5)	16,283 (99.8)
Yes	8,894 (30.2)	8,855 (67.5)	39 (0.2)
**Alcohol drinking**, ***n*** **(%)**			
No	22,536 (76.5)	7,879 (60.0)	14,657 (89.8)
Yes	6,915 (23.5)	5,250 (40.0)	1,665 (10.2)
**Vegetable intake**, ***n*** **(%), (times/week)**			
Tertile 1, <14	9,473 (32.2)	4,075 (31.0)	5,398 (33.1)
Tertile 2, ≥14 to <17.5	8,461 (28.7)	3,702 (28.2)	4,759 (29.1)
Tertile 3, ≥17. 5	11,517 (39.1)	5,352 (40.8)	6,165 (37.8)
**Fruit intake**, ***n*** **(%), (times/year)**			
Tertile 1, <2.4	10,462 (35.5)	3,960 (30.2)	6,502 (39.8)
Tertile 2, ≥2.4 to <24	8,609 (29.2)	3,850 (29.3)	4,759 (29.2)
Tertile 3, ≥24	10,380 (35.3)	5,319 (40.5)	5,061 (31.0)
**Education level, n (%)**			
Never	11,808 (40.1)	2,400 (18.3)	9,408 (57.6)
One to 5 years	9,190 (31.2)	5,848 (44.5)	3,342 (20.5)
Primary school	3,148 (10.7)	2,172 (16.5)	976 (6.0)
High school or higher education	2,705 (9.2)	2,097 (16.0)	608 (3.7)
Others	2,600 (8.8)	612 (4.7)	1,988 (12.2)
**Family history of cancer, n (%)**			
No	19315 (65.6)	8,500 (64.7)	10,815 (66.3)
Yes	10,136 (34.4)	4,629 (35.3)	5,507 (33.7)

Table 2 summaries the hazard ratios for the associations of the individual lifestyle factors and risk of total, heart disease, stroke and cancer mortality. Each of the unhealthy lifestyle factors: being underweight, overweight or obese, smoking, no alcohol intake, having a low vegetable intake, and having a low fruit intake, was associated with a significantly higher risk of all-cause mortality. As expected, there was more variability in the risks observed for the associations of the different health behaviors and cause-specific mortalities.

**Table 2:** Hazard ratios (HRs) and 95% confidence intervals (95% CIs) for the associations between individual lifestyle factors and risk of all-cause and cause-specific mortality.

**Lifestyle factors**	**All-cause**		**Heart disease**		**Stroke**		**Cancer**	
	**No. of death cases**	Multivariable HR (95% CI) ^*^	**No. of death cases**	Multivariable HR (95% CI) ^*^	**No. of death cases**	Multivariable HR (95% CI) ^*^	**No. of death cases**	Multivariable HR (95% CI) ^*^
**Body mass index, kg/m** ^**2**^								
<18.5	1,273	**1.12** **(1.05, 1.18)**	414	**1.34** **(1.21, 1.48)**	328	**0.87** **(0.77, 0.97)**	324	1.06 (0.95, 1.19)
≥18.5 to <25.0	16,981	1.00 (Ref)	4,128	1.00 (Ref)	5,311	1.00 (Ref)	5,162	1.00 (Ref)
≥25.0 to <30.0	2,088	**1.14** **(1.09, 1.19)**	458	1.03 (0.93, 1.13)	870	**1.47** **(1.37, 1.58)**	490	0.91 (0.83, 1.00)
≥ 30.0	124	**1.43** **(1.20, 1.70)**	26	1.12 (0.76, 1.65)	55	**1.87** **(1.43, 2.44)**	20	0.86 (0.56, 1.34)
**Smoking**								
Ever or current	6,900	**1.16** **(1.11, 1.21)**	1,673	**1.35** **(1.24, 1.48)**	1,913	1.02 (0.95, 1.10)	2,290	**1.20** **(1.11, 1.29)**
Never	13,566	1.00 (Ref)	3,353	1.00 (Ref)	4,651	1.00 (Ref)	3,706	1.00 (Ref)
**Alcohol drinking**								
Never	15,900	**1.13** **(1.03, 1.24)**	4,003	1.19 (0.97, 1.46)	5,206	0.98 (0.83, 1.16)	4,491	**1.31** **(1.10, 1.56)**
Ever or current	4,566	1.00 (Ref)	1,023	1.00 (Ref)	1,358	1.00 (Ref)	1,505	1.00 (Ref)
**Vegetable intake, times/week**								
Tertile 1, <14	6,753	1.02 (0.99, 1.06)	1,675	1.03 (0.96, 1.10)	2,158	1.00 (0.94, 1.06)	1,905	1.00 (0.94, 1.07)
Tertile 2, ≥14 to <17.5	5,795	1.00 (0.96, 1.03)	1,434	1.00 (0.94, 1.08)	1,880	1.00 (0.94, 1.06)	1,681	0.99 (0.93, 1.06)
Tertile 3, ≥17.5	7,918	1.00 (Ref)	1,917	1.00 (Ref)	2,526	1.00 (Ref)	2,410	1.00 (Ref)
**Fruit intake, times/year**								
Tertile 1, <2.4	7,943	**1.10** **(1.07, 1.14)**	2,127	**1.17** **(1.01, 1.25)**	2,536	**1.06** **(1.00, 1.13)**	2,172	1.05 (0.99, 1.12)
Tertile 2, ≥2.4 to <24	5,841	**1.05** **(1.02, 1.09)**	1,400	**1.09** **(1.01, 1.17)**	1,866	1.01 (0.95, 1.07)	1,704	1.02 (0.96, 1.09)
Tertile 3, ≥24	6,682	1.00 (Ref)	1,499	1.00 (Ref)	2,162	1.00 (Ref)	2,120	1.00 (Ref)

* *Adjusted for age at baseline, sex, commune, education level, and family history of cancer. Bold text indicates statistical significance*.

**Figure 1:**
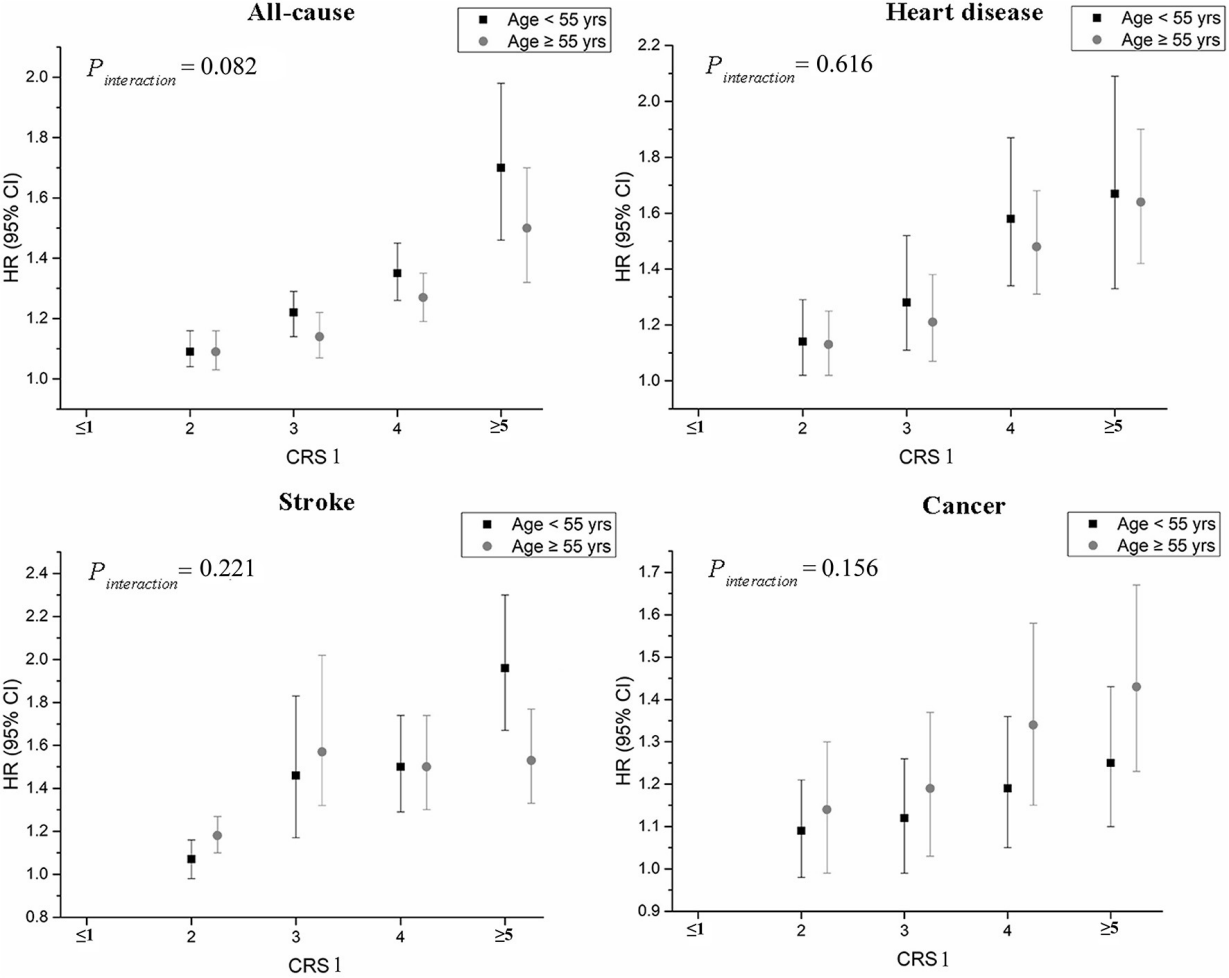
Hazard ratios and 95% confidence intervals for the associations between CRS1 and risk of all-cause and cause-specific mortality by age at baseline in the Linxian Nutrition Intervention Trial Cohort. Age at baseline was divided into two groups (<55 years and ≥55 years), based on the median value. Multivariable hazard ratios were adjusted for age at baseline, sex, commune, education level, and family history of cancer. CRS, combined risk score.

**Figure 2:**
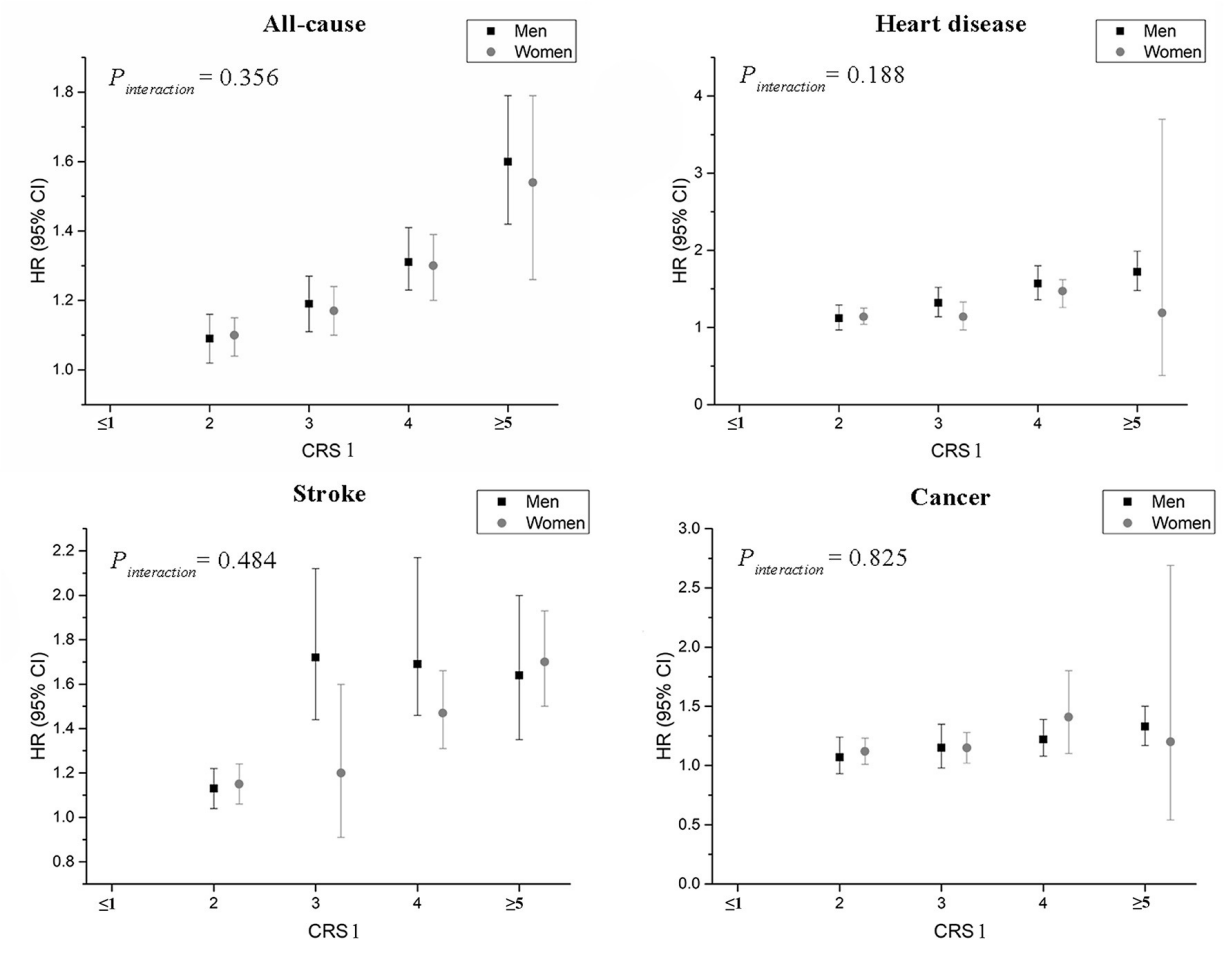
Hazard ratios and 95% confidence intervals for the associations between CRS1 and risk of all-cause and cause-specific mortality by men and women in the Linxian Nutrition Intervention Trial Cohort. Multivariable hazard ratios were adjusted for age at baseline, commune, education level, and family history of cancer. CRS, combined risk score.

Table 3 presents the HRs for the CRS1 of multiple lifestyle factors and overall and cause-specific mortality. As shown in the table, all categories of mortality elevated with an increasing risk score (all *P*_*trend*_ values < 0.05). As compared with subjects who had a CRS1 of 0–1 point, the HR for individuals with five or more points was 1.59 (95% CI: 1.44, 1.75) for all-cause mortality, 1.67 (1.48, 1.88) for heart disease mortality, 1.69 (1.52, 1.88) for stroke mortality and 1.34 (1.21, 1.47) for cancer mortality. Subgroup analyses by age at baseline indicated similar associations among younger (<55 years) and older participants (≥55 years). The strongest association was for deaths due to stroke among younger individuals who had a score of 5 or more points (HR = 1.96, 95% CI: 1.67, 2.30) (Figure 1). Results for men and women were similar to each other, with moderate increases in risk for all-cause, heart disease, and stroke mortality and relatively small associations for cancer mortality (Figure 2). Sensitivity analyses by exclusion of individuals who died the first 3 years of follow-up did not materially alter our results (Supplementary Figure 2). Similar patterns were generally seen for the association between CRS 2 and total, cause-specific mortality (Supplementary Table 3). Cumulative mortality curves of total, heart disease, stroke, and cancer by CRS1 categories are presented in Supplementary Figure 3.

**Table 3:** HRs and 95% CIs for the associations between CRS 1 and risk of all-cause and cause-specific mortality.

**Mortality**	HR and 95% CI^*^						***P_***trend***_***
	**Continuous**	**CRS1≤1**	**2**	**3**	**4**	**≥5**	
**All-cause**							
No. of deaths	20,466	3,398	7,995	4,809	3,793	471	
Age- and sex-adjusted HR (95%CI)	**1.10 (1.09, 1.12)**	1.00 (Ref)	**1.19 (1.05, 1.14)**	**1.18 (1.13, 1.23)**	**1.32 (1.26, 1.38)**	**1.60 (1.45, 1.76)**	<0.001
Multivariate adjusted HR (95%CI)^*^	**1.10 (1.08, 1.11)**	1.00 (Ref)	**1.10 (1.05, 1.14)**	**1.18 (1.13, 1.23)**	**1.31 (1.25, 1.37)**	**1.59 (1.44, 1.75)**	<0.001
**Heart disease**							
No. of deaths	5,026	978	2,154	660	764	470	
Age- and sex-adjusted HR (95%CI)	**1.15 (1.12, 1.18)**	1.00 (Ref)	**1.14 (1.05, 1.23)**	**1.26 (1.14, 1.40)**	**1.52 (1.38, 1.68)**	**1.74 (1.55, 1.96)**	<0.001
Multivariate adjusted HR (95%CI)^*^	**1.14 (1.11, 1.17)**	1.00 (Ref)	**1.13 (1.05, 1.22)**	**1.24 (1.18, 1.37)**	**1.51 (1.37, 1.67)**	**1.67 (1.48, 1.88)**	<0.001
**Stroke**							
No. of deaths	6,564	3,259	2,380	144	396	385	
Age- and sex-adjusted HR (95%CI)	**1.14 (1.12, 1.16)**	1.00 (Ref)	**1.12 (1.07, 1.19)**	**1.45 (1.23, 1.72)**	**1.49 (1.34, 1.66)**	**1.66 (1.49, 1.84)**	<0.001
Multivariate adjusted HR (95%CI)^*^	**1.15 (1.12, 1.17)**	1.00 (Ref)	**1.14 (1.08, 1.20)**	**1.51 (1.28, 1.79)**	**1.51 (1.36, 1.68)**	**1.69 (1.52, 1.88)**	<0.001
**Cancer**							
No. of deaths	5,996	867	1,870	1,054	921	1,284	
Age- and sex-adjusted HR (95%CI)	**1.09 (1.07, 1.11)**	1.00 (Ref)	**1.11 (1.02, 1.20)**	**1.19 (1.09, 1.31)**	**1.30 (1.18, 1.44)**	**1.41 (1.28, 1.56)**	<0.001
Multivariate adjusted HR (95%CI)^*^	**1.07 (1.05, 1.10)**	1.00 (Ref)	**1.10 (1.02, 1.19)**	**1.14 (1.04, 1.25)**	**1.25 (1.13, 1.38)**	**1.34 (1.21, 1.47)**	<0.001

*HR, hazard ratio; 95% CI, 95% confidence interval; CRS, combined risk score*.

* *Adjusted for age at baseline, sex, commune, education level, and family history of cancer. Bold text indicates statistical significance*.

## Discussion

In this large population-based cohort study, we found that being underweight, overweight or obese, smoking, no alcohol intake, having a low vegetable intake, and having a low fruit intake were independently associated with higher risk of all-cause, heart disease, stroke and cancer mortality. There was also a significant trend of increasing mortality risk with increasing values of the combined risk score. Significant associations were observed for total and cause-specific mortality, regardless of gender and age at baseline. After excluding subjects who were followed up <3 years, our results did not materially change.

In previous prospective cohort studies, smoking, overweight or obesity, and dietary patterns have consistently been associated with increased risk of chronic diseases (1, 2, 5). Not all of these risk factors could be studied in randomized controlled trials with end points of disease due to ethical or feasibility concerns. However, evidence from randomized controlled trials indicates that Mediterranean-style diet could be a protective factor of coronary heart disease (19). In addition, there is also evidence support the protective effect of a healthy diet, the combination of physical activity, and moderate weight loss for type 2 diabetes (20); and of smoking cessation for premature mortality (21).

A number of studies have demonstrated associations of heavy alcohol consumption with a higher risk of liver cirrhosis, stroke, coronary artery disease, various cancers, and hypertension (22, 23). Consistent with previous studies from Linxian, however, we found that alcohol consumption was associated with lower total and cancer mortality, and a nearly significant reduction in heart disease mortality as well. One possible reason for this was that alcohol consumption in Linxian, in 1985, was rare and quite modest, due to the expense of alcohol, and drinking alcohol was probably a marker for higher socioeconomic status (24). Indeed, one limitation of our study was the lack of information on the amount of alcohol consumption, which could have helped explain the observed mortality associations. In western studies, moderate alcohol consumption has been reported to have debated benefits, indicating either beneficial or harmful effects (25, 26). Nonetheless, determination of beneficial effects could be biased from healthy lifestyle behaviors, and harmful effects could also be overestimated due to related unhealthy factors, such as smoking, uncontrolled diet, and physical inactivity. Thus, the overall balance of beneficial and harmful effects of alcohol should be considered when making recommendations.

The effect of lifestyle factors such as smoking, alcohol drinking, BMI, and diet on health is overwhelming. Previous studies have also reported the protective effects of combinations of lifestyle factors on mortality (7–15, 27–30). Results from the Nurses' Health Study (NHS) of 77,782 women aged 34–59 years during 24 years of follow-up indicated that RRs for five lifestyle factors (being overweight, cigarette smoking, no light-to-moderate alcohol intake, taking little moderate-to-vigorous physical activity, and low diet quality score) were 4.31 for total mortality, 3.26 for cancer mortality, and 8.17 for cardiovascular mortality. They estimated that these lifestyle factors could be responsible for ~55% of the deaths occurring in this cohort (13). In addition, the US Health Professionals Study of 42,847 men aged 40–75 years, followed for 16 years, suggested that men with five healthy behaviors, including not smoking, BMI <25 kg/m^2^, moderate-to-vigorous activity, moderate alcohol consumption, and being in the top 40% of a healthy diet score, had an 87% lower risk of coronary heart disease compared with men who had none of these factors (31). More recently, Veronese et al. examined the combined associations of physical activity, diet, smoking, and moderate alcohol drinking with body weight on risk of mortality in the NHS and the Health Professionals Follow-up Study after 30-years follow-up. In each of the four categories of BMI studied, individuals who had healthy lifestyle factors had a significantly lower risk of total, cardiovascular, and cancer mortality as compared with individuals who had no healthy lifestyle factors. For BMI between 18.5–22.4, subjects with a combination of at least three healthy lifestyle factors had the lowest risk of all cause (HR = 0.39, 95% CI: 0.35, 0.43), cancer (0.40, 95% CI: 0.34, 0.47), and cardiovascular (0.37, 95% CI: 0.29, 0.46) mortality, as compared with those with BMI of 22.5–24.9 and none of the healthy lifestyle factors (12). Most of the previously published studies of the effects of multiple lifestyle factors on mortality have been conducted in western countries, but a few studies have examined these associations in Asian populations, including one study conducted in China (7) and three conducted in Japan (10, 32, 33). Each of these studies indicated that healthier lifestyles, defined by several lifestyle-related factors, were associated with substantial reductions in death in Asian populations, consistent with our findings.

Effective recommendations for preventing cardiovascular disease and cancer are very important for the public health. General recommendations for diet have been released which are supported by strong evidence. In addition, Scicchitano P et al. observed the impact of nutraceuticals in managing lipid disorders, which could play an important role in the occurrence of cardiovascular disease (34). However, the previous survey showed that because of the lower socio-economic status, dietary pattern in the Linxian population had the characteristics of single variety, and great seasonal effects (35), and the obtain of nutraceuticals was difficult. Therefore, the effect of nutraceuticals on the results was not evaluated in current study. Nonetheless, many people take dietary supplements, even though several studies have reported no effects of multivitamin supplementation on mortality or cardiovascular disease (36–38). These findings suggest that supplementing the diet with vitamin/mineral supplements has no clear benefit for well-nourished adults and may even be harmful, and that therefore these supplements should not be applied to chronic disease prevention in populations that do not have vitamin or mineral deficiencies.

### Strengths and Limitations

Our study had several important strengths, including its prospective design, large sample size, homogeneous ethnic makeup, and over 30 years of follow-up. Furthermore, we used a points system method to calculate the combined risk score of lifestyle factors, which was originally developed and validated by Sullivan et al. (18) based on The Framingham Heart Study. This method was a weighted approach based on the effect size of each factor which could improve the estimates of the overall impact of lifestyle factors on mortality. We also calculated a simple score that could be conceptually easy to understand and could be used in clinical practice and the development of public guidelines.

A number of limitations need to be noted regarding the present study. First, we only had a single baseline questionnaire to characterize individuals and thus could not take into account likely changes in lifestyles during the follow-up period, a time of dramatic changes in Chinese society, and this could contribute to misclassification of lifestyle factors. Second, there was no mention about the occurrence of well-known cardiovascular-specific risk factors such as hypertension, abnormal blood lipids, and adverse emotional states, which may impact results independently from lifestyle. Third, as noted above, no data were collected at baseline on the place and type of work of participants, air pollution, and the amount of alcohol, we cannot exclude the residual confounding due to these unmeasured factors. Fourth, although individuals who had ever been diagnosed with cancer or severe diseases (such as liver disease or severe kidney) were excluded from the main analyses, our results still may have been influenced by the presence of prevalent subclinical disease. Finally, our subjects were entirely composed of rural Chinese adults whose lifestyles and diets were assessed in 1985, which may affect the generalizability of our results to other populations today.

## Conclusions

In summary, we found that a higher combined risk score, based on five factors, was associated with risk of total, heart disease, stroke and cancer mortality. These results may indicate that even small differences in lifestyle may make a large difference to health. Future studies are needed to design appropriate interventions to reduce these unhealthy lifestyle factors in Asian populations.

## Data Availability Statement

The raw data supporting the conclusions of this article will be made available by the authors, without undue reservation.

## Ethics Statement

The studies involving human participants were reviewed and approved by Cancer Hospital, Chinese Academy of Medical Sciences (CHCAMS). The patients/participants provided their written informed consent to participate in this study.

## Author Contributions

Y-lQ, J-hF, PT, and CA: study concepts. Y-lQ, J-hF, PT, CA, and SD: study design. Y-lQ and J-hF: data acquisition and quality control of data and algorithms. J-bW and SD: data analysis and interpretation. HY and J-bW: statistical analysis and manuscript editing. J-hF, J-bW, and HY: manuscript preparation. HY, J-bW, J-hF, Y-lQ, PT, and CA: manuscript review. All authors contributed to the article and approved the submitted version.

## Funding

This work was supported by Key Project of Intergovernmental International Scientific and Technological Innovation Cooperation of National Key R&D Program (2021YFE0106000) and National Cancer Institute (USA), (https://www.cancer.gov/) grant numbers: N01-SC-91030 and N01-RC-47701.

## Conflict of Interest

The authors declare that the research was conducted in the absence of any commercial or financial relationships that could be construed as a potential conflict of interest.

## Publisher's Note

All claims expressed in this article are solely those of the authors and do not necessarily represent those of their affiliated organizations, or those of the publisher, the editors and the reviewers. Any product that may be evaluated in this article, or claim that may be made by its manufacturer, is not guaranteed or endorsed by the publisher.
