# Supplementary material for: Combined Lifestyle Factors and Risk of All-Cause and Cause-Specific Mortality Among Participants in the Linxian Nutrition Intervention Trial: A Cohort, Observational Study
Source: Front Cardiovasc Med. 2022 Feb 10;9:772617. doi: 10.3389/fcvm.2022.772617 (PMC8866236; doi:10.3389/fcvm.2022.772617)

Supplementary Material

Supplement 1

CRS 2 was created based on previous studies and public health recommendations [1-4]. To calculate the score, zero or one point was assigned to each of lifestyle factors, as follows: BMI (zero (≥18.5 to <25.0 kg/m^2) or one (<18.5 or ≥25.0 kg/m^2)) [5], smoking (zero (never) or one (ever)), alcohol drinking(zero (ever) or one(never)), vegetable intake(zero (highest tertile = ≥17.5 times/week) or one (lower two tertiles = <17.5 times/week)), and fruit intake(zero (highest tertile = ≥24 times/year) or one(lower two tertiles = <24 time/year)). For each factor, a score of one indicated an unhealthier lifestyle, and for each individual, their unhealthy lifestyle score was the sum of their scores for individual factor. For analysis, the CRS 2 was divided into five categories: one or less, two, three, four and five.

Cox proportional hazards regression models were used to calculate hazard ratios (HRs) and 95% confidence intervals (95% CIs) for the associations between CRS 2 and risk of all-cause and cause-specific mortality. This score was treated as a categorical variable and a score of one or less treated as the reference group in the Cox models.

eTables:

eTable 1. Assignment of CRS 2 in the Linxian General Population Nutrition Intervention Trial Cohort

eTable 2. Hazard ratios (HRs) and 95% confidence intervals (95% CIs) for the associations between CRS 2 and risk of all-cause and cause-specific mortality

eTable 3. Hazard ratios (HRs) and 95% confidence intervals (95% CIs) for the associations between CRS 2 and risk of all-cause and cause-specific mortality by age and gender

eTable 1. Assignment of CRS 2 in the Linxian General Population Nutrition Intervention Trial Cohort

Lifestyle factors	Scoring classification
**BMI (kg/m^2)**	
<18.5	1
≥18.5 to <25.0	0
≥25.0 to <30.0	1
≥ 30.0	1
**Smoking**	
Ever or current	1
Never	0
**Alcohol drinking**	
Ever or current	0
Never	1
**Vegetable intake (times/week)**	
Tertile 1, <14	1
Tertile 2, ≥14 to <17.5	1
Tertile 3, ≥17.5	0
**Fruit intake (times/year)**	
Tertile 1, <2.4	1
Tertile 2, ≥2.4 to <24	1
Tertile 3, ≥24	0

Abbreviation: CRS=combined risk score.

eTable 2. Hazard ratios (HRs) and 95% confidence intervals (95% CIs) for the associations between CRS 2 and risk of all-cause and cause-specific mortality

**Mortality**	**Score classification**				
	0-1	2	3	4	5
All-cause					
Age and sex adjusted HR (95% CI)	1.00	1.10 (1.05, 1.16)	1.17 (1.12, 1.22)	1.26 (1.20, 1.33)	1.65 (1.43, 1.92)
Multivariable HR (95% CI) *	1.00	1.11 (1.06, 1.16)	1.18 (1.13, 1.24)	1.28 (1.21, 1.35)	1.68 (1.45, 1.95)
Heart disease					
Age and sex adjusted HR (95% CI)	1.00	1.17 (1.06, 1.28)	1.29 (1.17, 1.41)	1.43 (1.28, 1.59)	1.85 (1.38, 2.48)
Multivariable HR (95% CI) *	1.00	1.17 (1.06, 1.29)	1.30 (1.18, 1.43)	1.45 (1.30, 1.62)	1.85 (1.38, 2.48)
Stroke					
Age and sex adjusted HR (95% CI)	1.00	1.08 (1.00, 1.17)	1.14 (1.05, 1.23)	1.23 (1.12, 1.35)	1.54 (1.16, 2.03)
Multivariable HR (95% CI) *	1.00	1.10 (1.01, 1.19)	1.16 (1.07, 1.26)	1.25 (1.14, 1.38)	1.55 (1.17, 2.05)
Cancer					
Age and sex adjusted HR (95% CI)	1.00	1.05 (0.97, 1.13)	1.09 (1.00, 1.18)	1.10 (1.00, 1.21)	1.54 (1.18, 2.02)
Multivariable HR (95% CI) *	1.00	1.04 (0.96, 1.13)	1.10 (1.01, 1.19)	1.11 (1.01, 1.23)	1.59 (1.21, 2.08)

Abbreviation: CRS=combined risk score.
*Adjusted for age at baseline, sex, commune, education level, and family history of cancer.

eTable 3. Hazard ratios (HRs) and 95% confidence intervals (95% CIs) for the associations between CRS 2 and risk of all-cause and cause-specific mortality by age and gender

Mortality	Score classification				
	0-1	2	3	4	5
All-cause					
Age <55 yrs*	1.00	1.08 (1.01, 1.14)	1.16 (1.10, 1.24)	1.26 (1.16, 1.37)	2.14 (1.64, 2.81)
Age ≥55 yrs*	1.00	1.14 (1.07, 1.22)	1.19 (1.11, 1.27)	1.28 (1.19, 1.38)	1.53 (1.28, 1.83)
Men #	1.00	1.14 (1.07, 1.22)	1.21 (1.13, 1.29)	1.30 (1.21, 1.40)	1.69 (1.45, 1.97)
Women #	1.00	1.07 (1.01, 1.14)	1.15 (1.08, 1.22)	1.23 (1.14, 1.34)	3.49 (1.12, 10.82)
Heart disease					
Age <55 yrs*	1.00	1.15 (0.99, 1.33)	1.29 (1.11, 1.49)	1.48 (1.23, 1.77)	2.50 (1.33, 4.72)
Age ≥55 yrs*	1.00	1.20 (1.06, 1.37)	1.33 (1.18, 1.51)	1.47 (1.28, 1.69)	1.75 (1.25, 2.44)
Men #	1.00	1.29 (1.12, 1.50)	1.42 (1.23, 1.63)	1.64 (1.41, 1.91)	2.02 (1.49, 2.75)
Women #	1.00	1.08 (0.95, 1.23)	1.22 (1.08, 1.39)	1.28 (1.09, 1.51)	- $
Stroke					
Age <55 yrs*	1.00	1.08 (0.96, 1.21)	1.14 (1.02, 1.27)	1.35 (1.17, 1.56)	2.09 (1.23, 3.57)
Age ≥55 yrs*	1.00	1.11 (0.99, 1.24)	1.17 (1.05, 1.31)	1.20 (1.05, 1.36)	1.39 (1.00, 1.94)
Men #	1.00	1.13 (1.00, 1.27)	1.17 (1.04, 1.31)	1.25 (1.10, 1.43)	1.57 (1.18, 2.09)
Women #	1.00	1.08 (0.97, 1.21)	1.17 (1.05, 1.30)	1.28 (1.11, 1.47)	- $
Cancer					
Age <55 yrs*	1.00	1.00 (0.91, 1.11)	1.05 (0.95, 1.17)	1.03 (0.90, 1.17)	1.73 (1.10, 2.70)
Age ≥55 yrs*	1.00	1.07 (0.93, 1.23)	1.09 (0.95, 1.25)	1.11 (0.95, 1.29)	1.44 (1.02, 2.03)
Men #	1.00	1.07 (0.96, 1.19)	1.12 (1.00, 1.25)	1.15 (1.01, 1.30)	1.55 (1.17, 2.05)
Women #	1.00	0.99 (0.88, 1.11)	1.00 (0.89, 1.13)	0.98 (0.83, 1.15)	6.95 (1.73, 27.91)

Abbreviation: CRS=combined risk score.
*Adjusted for age at baseline, sex, commune, education level, and family history of cancer.
#Adjusted for age at baseline, commune, education level, and family history of cancer.
$ HR and 95% CI cannot be calculated due to the small number of death cases.

References

1. Prospective Studies Collaboration, Whitlock G, Lewington S, et al. Body-mass index and cause-specific mortality in 900 000 adults: collaborative analyses of 57 prospective studies. Lancet. 2009; 373:1083-1096.
2. Wen W, Shu XO, Gao YT, et al. Environmental tobacco smoke and mortality in Chinese women who have never smoked: prospective cohort study. BMJ. 2006; 333: 376.
3. Steffen LM, Jacobs DR Jr, Stevens J, et al. Associations of whole-grain, refined-grain, and fruit and vegetable consumption with risks of all-cause mortality and incident coronary artery disease and ischemic stroke: the Atherosclerosis Risk in Communities (ARIC) Study. Am J ClinNutr. 2003; 78:383-390.
4. Genkinger JM, Platz EA, Hoffman SC, et al. Fruit, vegetable, and antioxidant intake and all-cause, cancer, and cardiovascular disease mortality in a community-dwelling population in Washington County, Maryland. Am J Epidemiol. 2004; 160:1223-1233.
5. WHO. Obesity: preventing and managing the global epidemic. Report of a WHO consultation on obesity. Geneva: WHO; 1997.

Supplement 2

eFigure 1. Consort Flow Diagram of the Linxian General Population Trial.

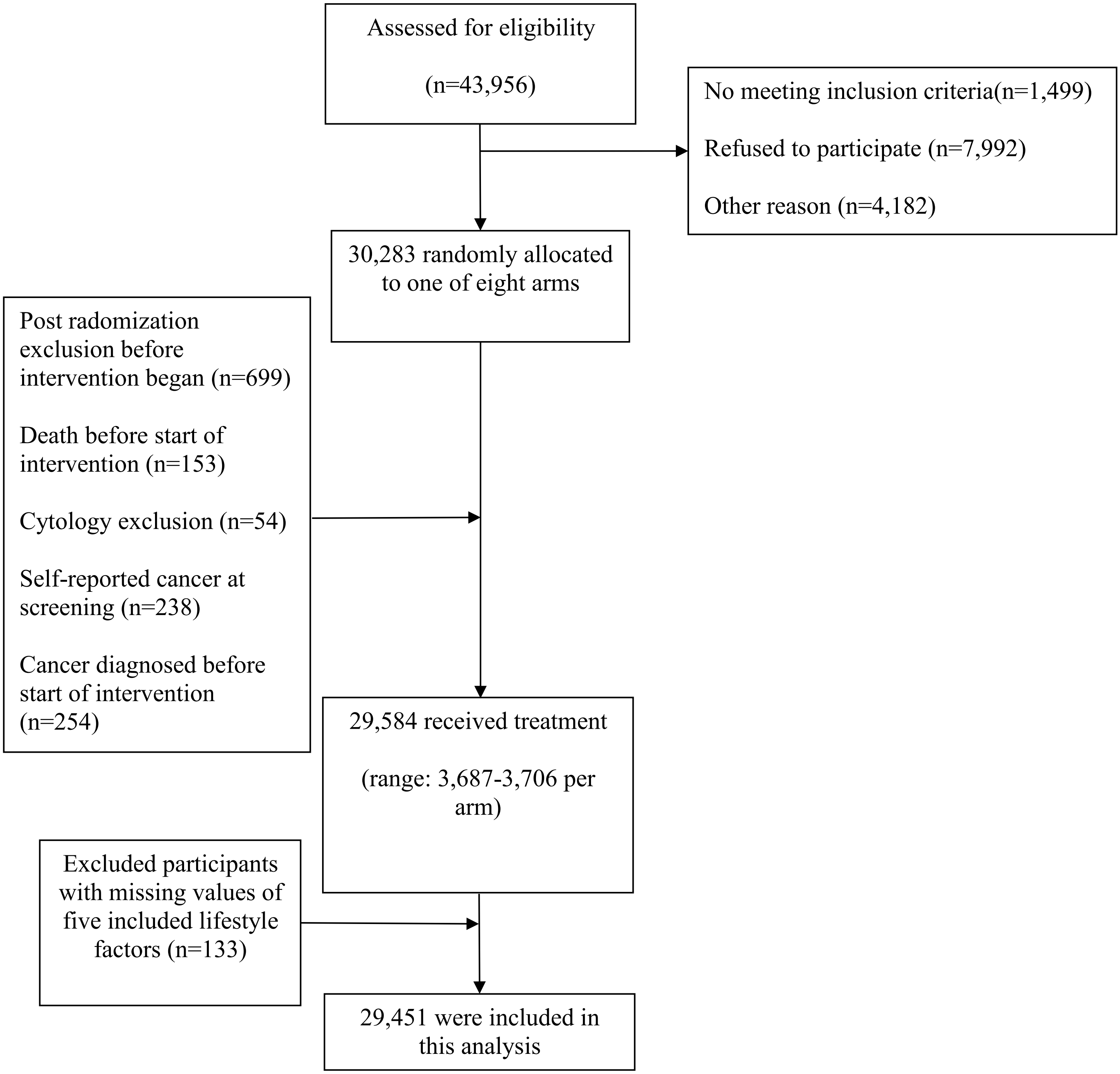

eFigure 2. Sensitivity analyses by exclusion of subjects with the first three years of follow up in the Linxian Nutrition Intervention Trial Cohort. Multivariable hazard ratios were adjusted for age at baseline, sex, commune, education level, and family history of cancer. Abbreviation: CRS 1=combined risk score 1.

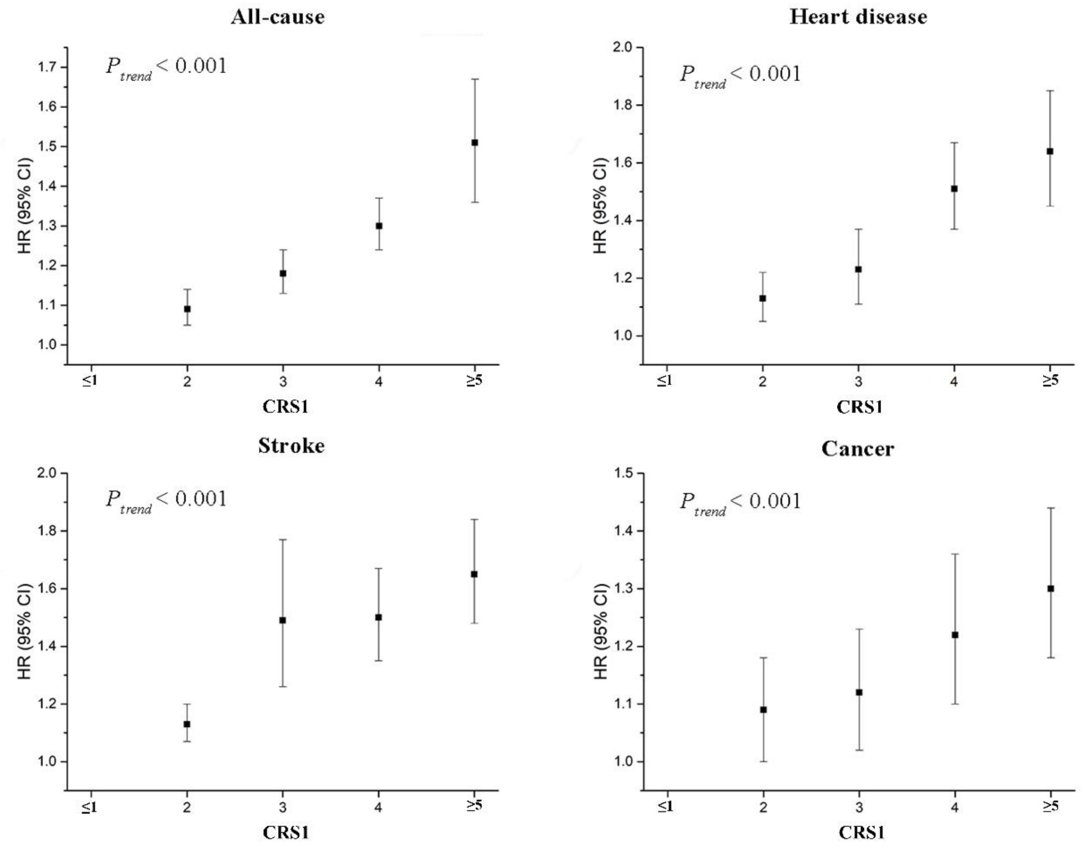

eFigure3. Cumulative mortality curves of all-cause, heart disease, stroke, and cancer mortality by CRS1 categories.

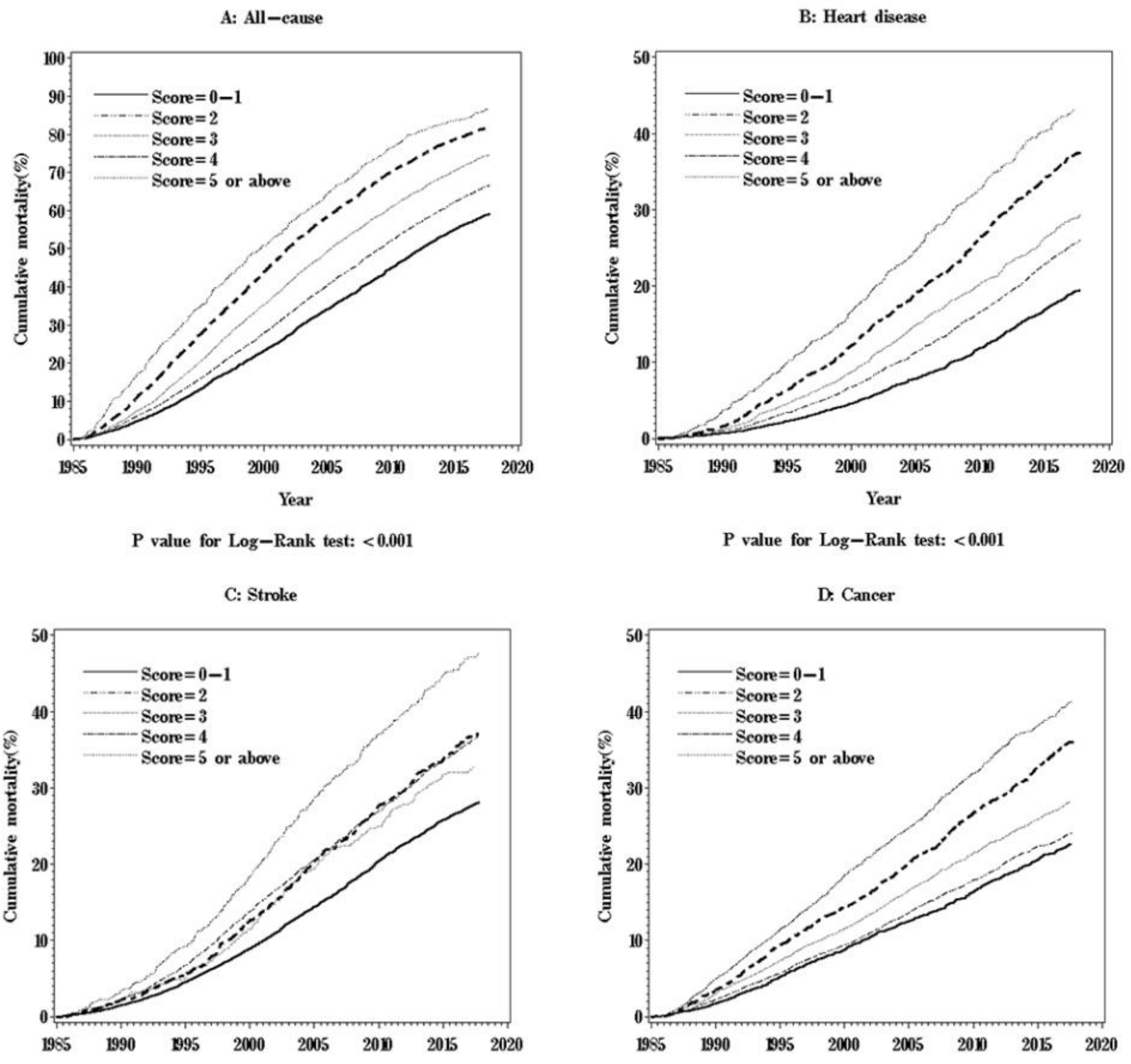

Supplement: Supplementary file 1 [file Data_Sheet_1.PDF]
